# High rate of postoperative upstaging of ductal carcinoma in situ when prioritizing ultrasound evaluation of mammography-detected lesions: a single-center retrospective cohort study

**DOI:** 10.1186/s12957-023-02900-6

**Published:** 2023-02-17

**Authors:** Yung-Chun Hsieh, Chiao Lo, Yi-Hsuan Lee, Ning Chien, Tzu-Pin Lu, Li-Wei Tsai, Ming-Yang Wang, Wen-Hung Kuo, Yeun-Chung Chang, Chiun-Sheng Huang

**Affiliations:** 1 National Taiwan University College of Medicine, Taipei, Taiwan; 2 Department of Surgery, National Taiwan University Hospital Hsin-Chu Branch, Hsin-Chu, Taiwan; 3 Department of Surgery, National Taiwan University Hospital, Taipei, Taiwan; 4 Department of Pathology, National Taiwan University Hospital, Taipei, Taiwan; 5 Department of Medical Imaging, National Taiwan University Hospital, Taipei, Taiwan; 6 Department of Epidemiology and Preventive Medicine, National Taiwan University College of Public Health, Taipei, Taiwan; 7 Department of Surgical Oncology, National Taiwan University Cancer Center, Taipei, Taiwan

**Keywords:** Breast cancer, Ductal carcinoma in situ, Sentinel lymph node biopsy, Ultrasonography, Mammography

## Abstract

**Background:**

The initial diagnosis of ductal carcinoma in situ (DCIS) can be upstaged to invasive cancer after definitive surgery. This study aimed to identify risk factors for DCIS upstaging using routine breast ultrasonography and mammography (MG) and to propose a prediction model.

**Methods:**

In this single-center retrospective study, patients initially diagnosed with DCIS (January 2016–December 2017) were enrolled (final sample size = 272 lesions). Diagnostic modalities included ultrasound-guided core needle biopsy (US-CNB), MG-guided vacuum-assisted breast biopsy, and wire-localized surgical biopsy. Breast ultrasonography was routinely performed for all patients. US-CNB was prioritized for lesions visible on ultrasound. Lesions initially diagnosed as DCIS on biopsy with a final diagnosis of invasive cancer at definitive surgery were defined as “upstaged.”

**Results:**

The postoperative upstaging rates were 70.5%, 9.7%, and 4.8% in the US-CNB, MG-guided vacuum-assisted breast biopsy, and wire-localized surgical biopsy groups, respectively. US-CNB, ultrasonographic lesion size, and high-grade DCIS were independent predictive factors for postoperative upstaging, which were used to construct a logistic regression model. Receiver operating characteristic analysis showed good internal validation (area under the curve = 0.88).

**Conclusions:**

Supplemental screening breast ultrasonography possibly contributes to lesion stratification. The low upstaging rate for ultrasound-invisible DCIS diagnosed by MG-guided procedures suggests that it is unnecessary to perform sentinel lymph node biopsy for lesions invisible on ultrasound. Case-by-case evaluation of DCIS detected by US-CNB can help surgeons determine if repeating biopsy with vacuum-assisted breast biopsy is necessary or if sentinel lymph node biopsy should accompany breast-preserving surgery.

**Trial registration:**

This single-center retrospective cohort study was conducted with the approval of the institutional review board of our hospital (approval number 201610005RIND). As this was a retrospective review of clinical data, it was not registered prospectively.

**Supplementary Information:**

The online version contains supplementary material available at 10.1186/s12957-023-02900-6.

## Background

The frequency of early detection of breast cancer has dramatically increased since the advent of mammography (MG) screening [1]. Various methods such as core needle biopsy (CNB), vacuum-assisted biopsy (VAB), or surgical excision under localization with ultrasonography (US) or MG are used for retrieving diagnostic specimens. Some cases of ductal carcinoma in situ (DCIS) can reportedly be “upstaged” to invasive cancer after definitive surgery. However, the current guidelines advise against performing sentinel lymph node biopsy (SLNB) along with breast-conserving surgery for cases with a preoperative diagnosis of DCIS [2]. The upstaging of DCIS to invasive cancer during definitive surgery may result in a second surgery for SLNB, which may fail because lymphatic drainage can be disrupted by previous excision [3, 4]; this necessitates axillary lymph node dissection, which is so undesirable that de-escalation of the axillary lymph node dissection procedure is also being studied [5].

Predicting postoperative upstaging of biopsy-diagnosed DCIS has been a popular topic among breast surgeons and radiologists. The rate of upstaging and associated risk factors varies according to previous studies. The reported postoperative upstaging rate ranged from 5% to 44% [6]. Although previous studies proposed various prediction models for DCIS upstaging, most of the models are hard to externally validate for general use [7–12].

In Taiwan, biennial screening mammography has been offered by the Health Promotion Administration, Ministry of Health and Welfare since 2004. It has facilitated the early detection of numerous lesions and frequent diagnosis of DCIS [13]. Thus, this study aimed to determine the upstaging rates, analyze the risk factors, and construct a prediction model for DCIS upstaging.

## Methods

**Fig. 1:**
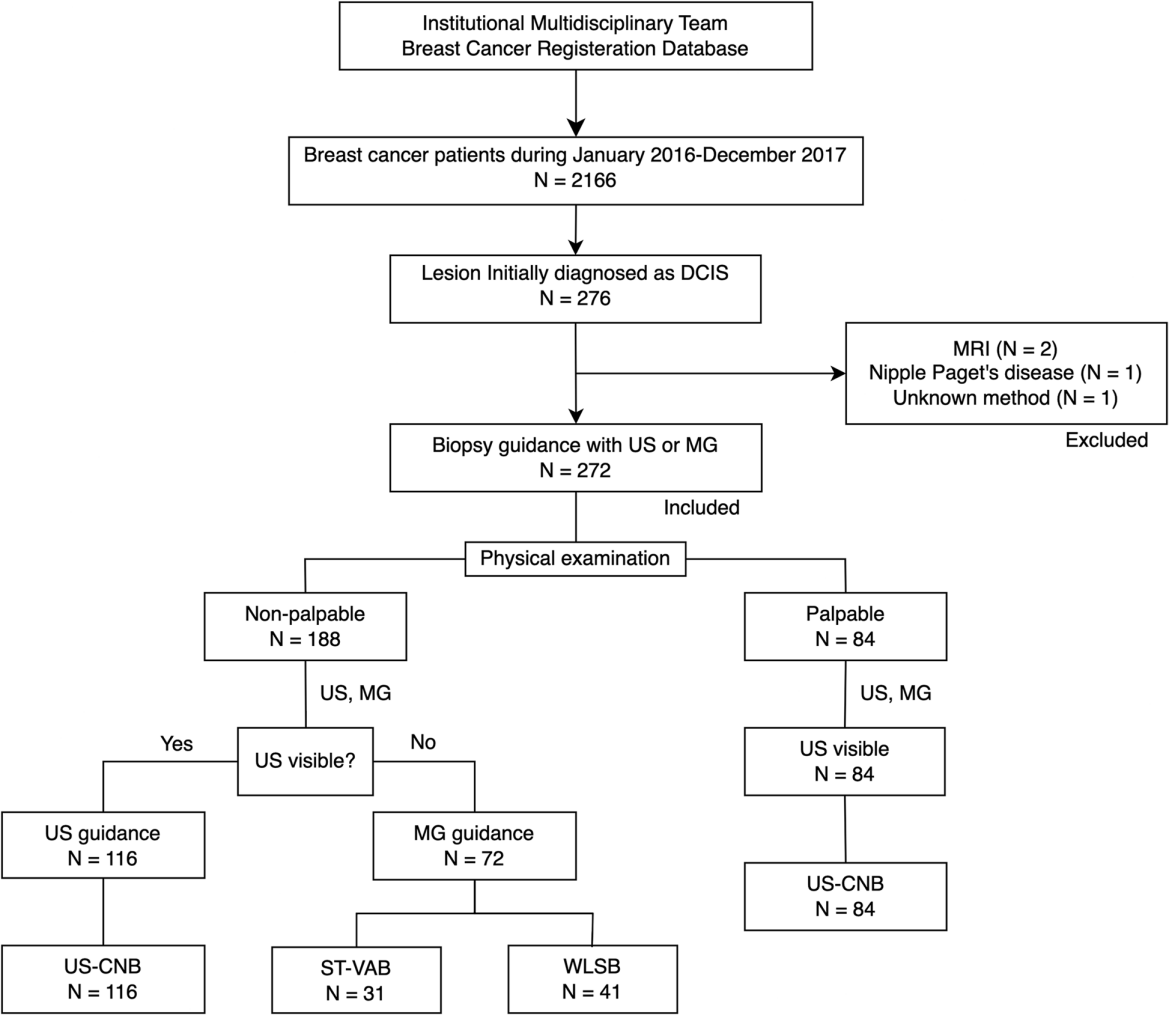
Patient selection chart and patient grouping. A diagram illustrating the inclusion and grouping of patients. DCIS, ductal carcinoma in situ; US, ultrasound; MG, mammography; US-CNB, ultrasound-guided core needle biopsy; ST-VAB, stereotactic vacuum-assisted breast biopsy; WLSB, wire-localized surgical biopsy

This single-center retrospective cohort study was conducted with the approval of the institutional review board of our hospital (approval number 201610005RIND). A total of 2166 newly diagnosed breast cancer cases were registered in our hospital’s Breast Cancer Multidisciplinary Team Database between January 2016 and December 2017. Patients with newly diagnosed DCIS of the breast before definitive surgery were enrolled in this study. Two patients who underwent magnetic resonance wire-localized excisions, one undergoing direct excision, and one undergoing nipple excision performed for Paget’s disease excision, were excluded. Thus, a total of 272 breast lesions that were initially diagnosed as DCIS were included in this study (Fig. 1).

### The routine diagnostic process and the selection of biopsy techniques

Routinely, physical examination was performed by a breast surgeon during clinic visit. All palpable lesions were evaluated by US, and all these patients’ diagnoses were made by US-CNB. For nonpalpable lesions, both US and MG were performed due to the high prevalence of dense breasts. The biopsy procedure was selected based on whether the lesion was visible on US. In Taiwan, the National Health Insurance (NHI) system pays for US, CNB, and surgical excision, but not for the stereotaxis technology and VAB systems. The out-of-pocket expense for the patient is approximately NT $5000 (USD $168) for wire-localized surgical biopsy (WLSB) and around NT $22,000 (USD $740) for MG-guided stereotactic VAB (MG-VAB). If a suspicious lesion was correlated between US and MG, US-CNB was usually prioritized due to the coverage by NHI system. For suspicious lesions only visible on MG, the biopsy choice was made between MG-VAB and WLSB (Fig. 1). The indication for upfront WLSB is either the patient has too thin breasts at MG compression, too wide area of suspicious microcalcifications, or the patient’s preference considered at patient’s expense.

### Diagnostic methods

#### Ultrasound-guided core needle biopsy

Bilateral whole-breast US examination was performed using a linear broadband transducer (Toshiba Aplio XG Model SSA-790 A at 7–18 MHz [Toshiba, Tokyo, Japan]; Hitachi Hi Vision Avius® at 5–13 MHz [Hitachi, Tokyo, Japan]; or Philips, Best, Netherlands iU22 xMATRIX at 5–12 MHz [Philips]) by certified technicians, all of whom had >5 years of experience. The images were interpreted by breast surgeons or radiologists, and BI-RADS category 4 or 5 lesions were indicated for US-guided percutaneous CNB (BARD® MAX-CORE® Disposable Core Biopsy Instrument [Becton Dickinson, Franklin Lakes, NJ]; Argon Medical SuperCore™ Semi-Automatic Biopsy Instrument [Argon Medical, Frisco, TX]; or Merit Medical Temno Evolution® Biopsy Device [Merit Medical, South Jordan, UT]). Routinely, 3–6 cores were obtained using a 16-G core needle.

#### Mammography-guided stereotactic vacuum-assisted biopsy

All patients underwent MG-guided stereotactic VAB (MG-VAB) that was performed by a radiologist using an upright add-on stereotactic unit (Delta DS Stereotaxy; GE Healthcare, Buc, France) interfaced with a digital MG machine (Senographe Essential, GE Healthcare, Buc, France), a 10-G SenoRxEncor Probe (SenoRx, Tempe, AZ), and an Enspire biopsy system (SenoRx). At least 12 specimens were retrieved for each target. After confirming the acquisition of the targeted microcalcifications from specimen radiography, a 2-mm metallic clip (Gel Mark Ultra Breast Tissue Marker, SenoRx) was introduced to mark the biopsy site. All patients underwent immediate post-biopsy MG with craniocaudal and mediolateral oblique views.

#### Wire-localized surgical biopsy

Magnified MG (Senographe Essential, GE Healthcare) was performed preoperatively to confirm the lesion location. The radiologist used a 7- or 9-cm localization needle (Ghiatas Beaded Breast Localization Wire, Becton Dickinson) to wire localize the lesion. Subsequently, a wide excision was made to excise the targeted lesion with the wire guidance. Specimen radiography was performed intraoperatively to ensure removal of the target lesion.

### Data review and coding

Patient medical records retrieved from the institutional electronic medical record system were reviewed by a surgeon who was not involved in the diagnosis and treatment. Coding and analysis were performed for each lesion. A diagnosis of bilateral DCIS in a single patient was coded as two cases. Patients with ipsilateral multifocal lesions were coded as single cases. The final staging was coded for each breast. Mammographs were reviewed by a breast radiologist, who was blinded to patient information and final staging results, based on the American College of Radiology Breast Imaging Reporting and Data System (BI-RADS). Biopsy and surgical specimens were examined by a breast pathologist. DCIS lesions were graded using the Van Nuys prognostic index [14].

### Surgical staging and outcome definition

The surgical pathology was evaluated by a breast pathologist. The final staging of the lesions was based on the 7^th^ edition American Joint Committee on Cancer criteria [15]. Lesions initially diagnosed as DCIS on biopsy with a final diagnosis of invasive cancer at definitive surgery were defined as “upstaged.” For the WLSB group, the biopsy included DCIS lesions having adequate margins that did not require further surgery and were categorized as “non-upstaged.” The DCIS lesions not having enough margins all underwent second wide excision surgery. If the final pathology is pure DCIS, it is also categorized as “non-upstaged.” If the second re-excision surgery diagnosed invasive cancer, it is categorized as “upstaged.”

### Statistical analysis

Numerical variables are expressed as mean and standard deviation. The Fisher’s exact test or chi-square test was used for categorical variables, and the Student’s *t*-test was used for numerical variables. Risk factor analysis was performed using logistic regression. Multiple logistic regression analysis was used to adjust confounders while risk factors were identified. Statistically significant variables were included in the prediction model. A best-fitting prediction model was constructed using best subsets regression analysis. Internal validation of prediction models was conducted by calculating the area under the receiver operating characteristic (ROC) curve (c-statistic). The final model selection was based on the c-statistic of the model, clinical significance, and clinical reproducibility of variables. The final model was depicted as a nomogram. Statistical significance was defined as *P* values <0.05 in a two-tailed test. Statistical analyses and plotting were performed using R 4.0.3 (R Foundation, Vienna, Austria).

## Results

Overall, 272 lesions with initial diagnoses of DCIS were extracted from the breast cancer registration database. We enrolled 263 women, nine of whom had bilateral lesions. The participants’ mean age was 54.0 ± 9.95 years and their body mass index (BMI) was 22.8 ± 3.8 kg/m^2^. While 30.9% (84/272) and 6.3% (17/272) patients had presented with the chief complaints of palpable lesions and nipple discharge, respectively, 62.9% (171/272) had been referred because of abnormalities detected without any subjective symptoms during screening examinations.

Patient characteristics, diagnostic methods, and lesion-associated variables are presented in Table 1. A total of 200, 31, and 41 DCIS lesions were diagnosed using US-CNB, MG-VAB, and WLSB, respectively. The patients’ age, BMI, history of breast cancer, and family history of breast cancer did not differ among the three diagnostic groups. The US-CNB group had a greater degree of palpability (*P*<0.001), US visibility (*P*<0.001), mass visibility on MG (*P*<0.001), and histological suspicion of microinvasion (*P*=0.003). Estrogen receptor (ER) positivity was lower in the US-CNB group than in the MG-VAB and WLSB groups (*P*=0.005).

**Table 1:** Comparison of patient characteristics, examination/diagnostic factors, and pathology among the diagnostic groups

Variables	Overall	US	MG		***P***^**a**^
	*N* = 272	US-CNB (*n* = 200)	MG-VAB (*n* = 31)	WLSB (*n* = 41)	
**Patient characteristics**					
Age	272				0.16
<50 years		81	7	16	
≥50 years		119	24	25	
BMI	269				0.13
<23.5		131	15	24	
≥23.5		66	16	17	
Previous history of breast cancer	272				1
No		180	28	37	
Yes		20	3	4	
Family history of breast cancer	248				0.46
No		144	19	31	
Yes		40	8	6	
Lesion side	272				0.69
Left		99	18	21	
Right		101	13	20	
**Diagnostic/examination factors**					
Initial detection method	272				< 0.001
Screening imaging		100	30	41	
Palpated by the patient		84	0	0	
Nipple discharge		16	1	0	
Palpability (by surgeon)	272				< 0.001
No		91	31	41	
Yes		109	0	0	
Lesion under US	261				< 0.001
Not detectable		0	26	32	
Detectable		200	1	2	
MG BI-RADS category^b^	210				0.005
1, 2, or 3		20	1	0	
0, 4, or 5		118	30	41	
MG mass or architectural distortion	207				< 0.001
No		87	29	31	
Yes		53	0	7	
**DCIS-associated factors (at biopsy)**					
DCIS grade	206				0.26
Low		21	8	6	
Intermediate		58	13	15	
High		66	8	11	
Suspicion of microinvasion	261				0.03
No		173	31	40	
Yes		17	0	0	
ER	217				0.005
Negative		52	3	3	
Positive		108	20	31	
**Definitive surgery type**	272				< 0.001
Breast conserving surgery		92	22	37	
Mastectomy		108	9	4	
**Diagnosis at definitive surgery**	272				< 0.001
DCIS		59	28	39	
Invasive cancer (upstage %)		141 (70.5)	3 (9.7)	2 (4.9)	

*Abbreviations*: *ER* estrogen receptor, *US-CNB* ultrasound-guided core needle biopsy, *MG-VAB* mammography-guided vacuum-assisted biopsy, *WLSB* wire localized surgical biopsy, *US* ultrasound, *MG* mammogram, *BI-RADS* The Breast Imaging and Data System, *DCIS* ductal carcinoma in situ, *BMI* body mass index

^a^Pearson’s chi-square test applied to the US and MG groups

^b^Excluded patients who underwent MG after US-CNB-proven DCIS (BI-RADS “6”)

The upstaging rates were 70.5% (141/200), 9.7% (3/31), and 4.9% (2/41) in the US-CNB, MG-VAB, and WLSB groups, respectively (*P*<0.001). The upstaged lesions in the US-CNB group (*n*=200) were staged as follows: 95.7% (135/141) as stage I disease (microinvasion: 72/135 = 53%) and 4.2% (6/141) as stage II disease. All five upstaged lesions in the MG-guided group (*n*=72) were staged as pT1 (two pT1mic, one pT1a, and two pT1b), and none of the lesions exhibited axillary lymph node metastasis. During the first intent-to-treat procedure, SLNB was performed for 183 lesions in the US-CNB group (91.5%, 183/200), 14 lesions in the MG-VAB group (54.8%, 17/31), and eight lesions in the WLSB group (19.5%, 8/41). After the first procedure without SLNB, six invasive cancers from the US-CNB group and three from MG-guided groups respectively underwent a second SLNB procedure after confirming the invasive nature of the lesion; they were all negative for lymph node metastases (although one presented with isolated tumor cells).

The comparisons of patient characteristics, examination/diagnostic factors, and pathological factors between the MG-VAB and WLSB groups are presented in Additional file 1. There were no differences in the upstaging rate, patient characteristics, examination factors, and pathological factors between the two groups, except for a higher frequency of MG mass-associated findings in the WLSB group (*P*=0.004); this could be ascribed to the surgeon’s preference. Among DCIS lesions identified using MG-guided procedures, one lesion diagnosed using MG-VAB and two lesions diagnosed using WLSB were visible on breast US. US-CNB was first obtained for these three lesions, and these were considered as image-pathology discordance by the surgeon. Subsequent MG-VAB or WLSB targeting suspicious microcalcifications confirmed the DCIS diagnosis for the three lesions.

Table 2 shows the logistic regression analysis results of risk factors for upstaging. The significant predictive factors included palpability (*P*<0.001), US-determined lesion size (*P*<0.001), MG BI-RADS category, MG mass lesion, use of US-CNB as the diagnostic method (*P*<0.001), histologically high-grade DCIS (*P*<0.001), suspicion of microinvasion (*P*=0.009), and Negative ER (*P*=0.02). Multiple logistic regression adjustment revealed that the use of US-CNB as the diagnostic method was the sole independent significant predictive factor (adjusted odds ratio [OR] = 2.6, *P*=0.02) for upstaging.

**Table 2:** Upstaging risk factor analysis for the general variables in all patients (*N* = 272)

Variables		Logistic regression			Multiple logistic regression		
	*n*	OR	95% CI	*P*	OR	95% CI	*P*
**Patient characteristics**							
Age	272	0.997	0.97–1.02	0.80			
BMI	269	0.98	0.92–1.04	0.48			
Previous history of breast cancer	272	0.8	0.3–1.7	0.54			
Family history of breast cancer	272	0.9	0.5–1.6	0.62			
Side = right	272	1.2	0.7–1.9	0.54			
Palpability	272	7.6	4.3–13.7	<0.001	2.2	0.7–6.6	0.16
**US and MG variables**							
US lesion size (cm)	246	3.8	2.7–5.6	<0.001	1.6	0.8–3.1	0.18
MG BI-RADS category (4, 5)	229	0.4	0.2–0.7	<0.001	1.2	0.4–3.9	0.80
MG mass or distortion	207	2.9	1.5–5.5	0.001	1.5	0.5–4.8	0.44
MG calcification	210	0.6	0.3–1.05	0.07	1.4	0.4–4.5	0.60
**DCIS histological characteristics**							
Diagnostic method (US-CNB)	272	32.0	13.5–95.0	<0.001	10.3	2.0–64	0.007
High DCIS grade	206	3.4	1.9–6.1	<0.001	2.2	0.8–6.6	0.16
Suspicious of microinvasion	261	15.2	3.0–277.0	0.009	3.8	0.6–75	0.24
Negative ER	217	2.1	1.1–4.0	0.02	1.1	0.4–3.2	0.92

*Abbreviations*: *OR* odds ratio, *CI* confidence interval, *BMI* body mass index, *US* ultrasound, *MG* mammogram, *US-CNB* ultrasound-guided core needle biopsy, *DCIS* ductal carcinoma in situ, *ER* estrogen receptor, *BI-RADS* The Breast Imaging and Data System

Univariable analysis results of the US-CNB subgroup are presented in Table 3. Palpability (OR = 3.0, *P*<0.001), US lesion size (OR = 2.1, *P*<0.001), MG calcification (OR=2.2, *P*=0.04), and high-grade DCIS (OR=4.8, *P*<0.001) were significant predictors for upstaging. These significant factors were included in the multiple logistic regression model. Palpability (adjusted OR=2.6, *P*=0.04) and US lesion size (adjusted OR=1.8, *P*=0.04) retained their significance after adjustment.MG calcification and histologically high-grade DCIS were correlated with each other.

**Table 3:** Upstaging risk factor analysis for US-CNB-diagnosed DCIS (*N* = 200)

Variables		Logistic regression			Multiple logistic regression		
	*n*	OR	95% CI	*P*	OR	95% CI	*P*
**US variables**							
Palpability	200	3.0	1.6–5.7	<0.001	2.3	0.9–6.2	0.1
Lesion size (cm)	185	2.1	1.4–3.3	<0.001	1.73	0.98–3.4	0.08
BI-RADS category (5)	176	3.6	0.97–23.3	0.10			
US-detected calcification	170	1.6	0.7–4.3	0.30			
**Associated MG variables**							
BI-RADS category (0, 4, 5)	157	1.5	0.7–3.2	0.30			
Mass or distortion	141	0.98	0.5–2.0	0.95			
Calcification	143	2.2	1.04–4.6	0.04	1.6	0.6–4.2	0.3
**Biopsy DCIS pathologic variables**							
Core biopsy strip number	181	1.2	0.95–1.5	0.16			
High DCIS grade	145	4.8	2.2–11.6	<0.001	2.6	0.98–7.5	0.06
Suspicious for microinvasion	190	7.1	1.4–129	0.06	4.0	0.6–77	0.2
Negative ER	160	1.34	0.6–3.0	0.45			

*Abbreviations*: *OR* odds ratio, *CI* confidence interval, *BI-RADS* The Breast Imaging Reporting and Data System, *CNB* core needle biopsy, *DCIS* ductal carcinoma in situ, *ER* estrogen receptor

As per the risk factor analysis, there was no significant factor associated with upstaging in the MG-guided group (Table 4). There was no difference in the upstaging rate between the MG-VAB and WLSB groups (9.7% versus 4.9%, *P*=0.65). No variable was observed to be significantly associated with upstaging in this group.

**Table 4:** Upstaging risk factor analysis for ductal carcinoma in situ diagnosed using mammography (MG)-guided procedures (*N* = 72)

Variables	Overall	DCIS (***n*** = 67)	Invasive cancer (%) ***(n*** = 5)	***P***^**a**^
**MG-associated factors**				
BI-RADS category	72			0.72
3		1	0	
4		9	0	
4a		16	1 (5.9)	
4b		28	4 (12)	
4c		11	0	
5		2	0	
Breast density	72			>0.99
B		1	0	
C		63	5	
D		3	0	
Mass or distortion	67			0.43
No		56	4 (6.7)	
Yes		6	1 (14)	
MG calcification	67			>0.99
No		2	0	
Yes		61	4 (6.2)	
**Biopsy DCIS factors**				
Diagnostic method	72			0.65
ST-VAB		28	3 (9.7)	
WLSB		39	2 (4.9)	
DCIS grade	61			>0.99
Low		13	1	
Intermediate		27	1	
High		18	1	
Suspicion of microinvasion	71			(NA)
No		66	5 (7.0)	
Yes		0	0	
ER	57			>0.99
Negative		6	0	
Positive		46	5 (9.8)	

*Abbreviations*: *BI-RADS* The Breast Imaging Reporting and Data System, *US-CNB* ultrasound-guided core needle biopsy, *ST-VAB* stereotactic vacuum-assisted breast biopsy, *WLSB* wire-localized surgical biopsy, *US* ultrasound, *MG* mammogram, *DCIS* ductal carcinoma in situ, *ER* estrogen receptor

^a^Fisher’s exact test

**Fig. 2:**
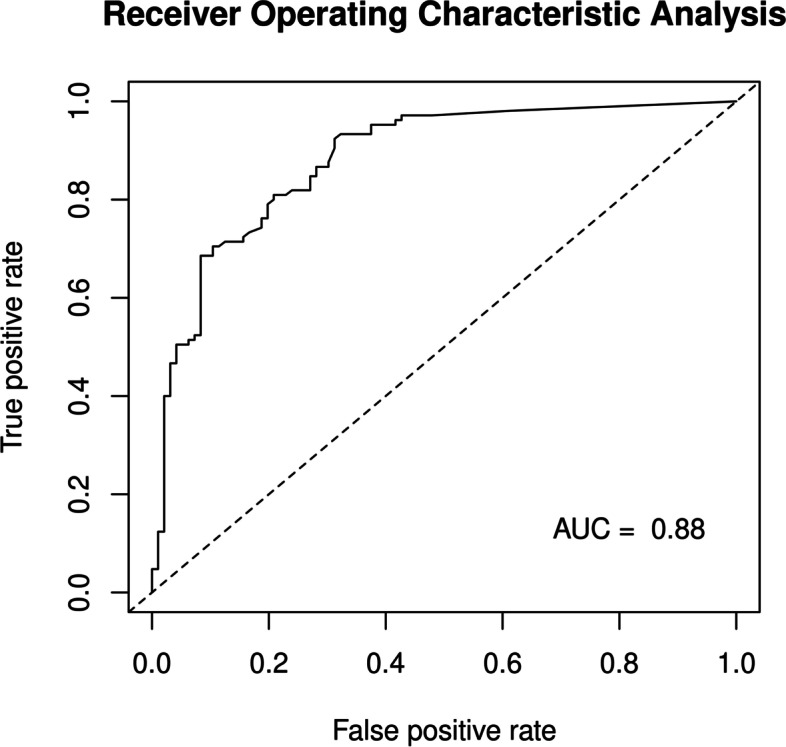
Receiver operating characteristic analysis. Receiver operating characteristic analysis of the final multiple logistic regression model (Table 5) with an AUC of 0.88. ROC, receiver operating characteristic; AUC, area under curve

**Fig. 3:**
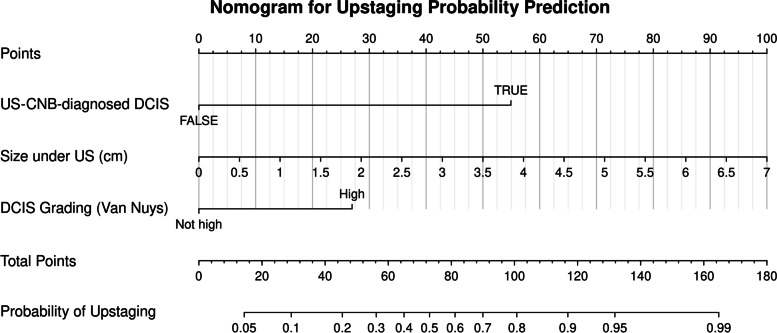
Nomogram for the multiple logistic regression prediction model. Nomogram for the probability of postoperative upstaging among preoperatively diagnosed DCIS. The value of each factor corresponds to “Points” vertically at the top scale. The “Points” for each factor are added together as “Total Points,” which vertically correspond to the “Probability of Upstaging” scale below. US, ultrasound; CNB, core needle biopsy; DCIS, ductal carcinoma in situ

The best-fitted model was constructed using all possible subset regression approaches and included identified independent variables. The best-fitted model could be constructed with four variables: “palpability,” “US lesion size,” “US-CNB diagnostic method,” and “high-grade DCIS.” The model performance was measured using the area under the curve (AUC), which yielded a value of 0.89. In this model, “palpability” and “US lesion size” confounded each other. The second best-fitting models were three-variable models, which excluded either “palpability” or “US lesion size.” Both models exhibited similar performance measurement results (AUC = 0.88). Given that “palpability” is a subjective evaluation, “US lesion size” was selected for best-fitting model construction owing to its superior clinical reproducibility. Therefore, “US lesion size,” “US-CNB diagnostic method,” and “high-grade DCIS” were used to construct the final model (Table 5). Figure 2 shows the ROC curve of this model. The nomogram for model visualization is presented in Fig. 3.

**Table 5:** Multiple logistic regression prediction model^a^

Variables	Coefficient	SE^**a**^	OR^**a**^	95% CI^**a**^	***P***
US lesion size	0.72	0.25	2.1	1.3–3.4	0.003
Biopsy method (US-CNB)	2.75	0.73	15.7	4.2–78.8	< 0.001
High DCIS grade	1.35	0.40	3.9	1.8–8.9	< 0.001

*Abbreviations*: *OR* odds ratio, *CI* confidence interval, *SE* standard error, *US* ultrasound, *CNB* core needle biopsy, *DCIS* ductal carcinoma in situ

^a^Adjusted for SE, OR, CI, and *P*

## Discussion

In this study, the postoperative upstaging rate for US-CNB-diagnosed DCIS was significantly higher than the overall upstaging rate. To the best of our knowledge, the upstaging rate of US-CNB-diagnosed DCIS reported in this study is the highest reported in literature at 70.5%. The reported upstaging rates of DCIS diagnosed using MG-VAB and WLSB (9.68% and 4.87%, respectively) are comparable with or lower than those reported in previous studies [6, 7, 9–11, 16, 17]. Our final model concluded “US-CNB,” “US lesion size,” and “high-grade DCIS” as the major risk factors of postoperative upstaging of DCIS lesions, which are similar to the model created by Jakub et al. [10].

The disparity between the upstaging rates of US-CNB and MG-guided procedures (MG-VAB and WLSB) observed in this study is the highest among available studies. The internal validation AUC of our model was 0.88, whereas the internal validation AUC of previously reported prediction models ranged from 0.62 to 0.75 (Additional file 2) [7–12]. The marked discrepancy of upstaging rates between biopsy techniques and high overall model AUC are possibly attributable to the sorting effect of the routine supplemental breast US on visualization of any suspicious focus on MG. High model AUC reflects a unified diagnostic process; however, it is not suggestive of predictions for those who do not follow the same process. Supplemental screening breast US is widely performed in Taiwan because of the high proportion of dense breasts. In this study, 98.6% (206/209) of the breasts were reported as composition “C” or “D.” A recent meta-analysis concluded that the sensitivity of screening MG with supplemental US was significantly higher than the screening MG alone (96% vs. 74%) in women with dense breasts, while the specificity was lower (87% vs. 93%) [18].

On the other hand, studies have provided evidence of the lower underestimation rate of VABs compared with that of CNBs under the same guidance modality [16, 19, 20]. From the perspective of lowering the postoperative upstaging rate, the use of VAB for small or non-mass-like lesions seems to be a reasonable choice. However, it adds up a considerable amount to the medical system cost. The use of VAB for US-detected small mass lesions could cause difficulty in reporting the actual histological size of small carcinomas [21].

In Taiwan, a universal breast cancer screening program has been implemented for women aged 50-69 years since 2004 and expanded to women aged 45-69 years since 2009. The Health Promotion Administration pays for a biennial screening MG. However, although a study has pointed out the better cost-effectiveness of stereotactic VAB for nonpalpable breast lesions, only US-CNB is covered by the National Health Insurance [22]. MG-guided stereotactic localization techniques and VAB procedures usually require an additional NT$5000 to NT$22,000 (around USD $168 to USD $740) at the expense of patients who are confronted with a suspicious finding at MG screening that may require further histological evidence. This policy not only affected the willingness for further examination but also the clinical diagnostic methods and treatment preference [23]. Although the retrospective cohort is from 2016 to 2017, the policy has persisted to date.

Invasive carcinoma in DCIS background frequently present as microcalcifications within an US-visible mass or those appearing on US in an ill-defined hypoechoic background [24, 25]. The use of supplemental US as a guiding modality, with automated core needle gun as biopsy device, amplified the underestimation rate of these lesions. As the SLNB procedure is covered by the NHI system, the concern of postoperative upstaging also adds to the more unnecessary SLNB procedures for patients with pure DCIS. Although there is no extra expense for patients if SLNB is performed in the second surgery, some patients prefer undergoing SLNB with the first breast-conserving surgery to avoid repeated queuing, admission, general anesthesia, surgery, and recovery. In our cohort, a total of 33 patients with a final diagnosis of DCIS underwent unnecessary SLNB accompanying breast-conserving surgery. Symptomatic lymphedema was not recorded in any of these patients within two years of surgery. However, this does not mean that there is no lymphedema at all. Since the prevalence of SLNB-related lymphedema is low, it usually requires a prospective study design with preoperative and postoperative arm circumference measurements to establish a consistent diagnosis.

The lack of US-visible DCIS diagnosed with MG-guided procedures is one of the limitations of this study. The retrospective study design rendered the comparison among studies difficult because the clinical diagnostic flow may directly affect the inclusion of biopsy-diagnosed DCIS cases.

## Conclusions

Our study findings suggest that it is unnecessary to perform SLNB during the first surgical treatment for MG-VAB- or WLSB-proven DCIS lesions that are not visible on US. For DCIS detected using US-CNB, case-by-case evaluation is essential to determine whether it is necessary to repeat a biopsy with VAB or if it is feasible to proceed SLNB with wide excision.

## Supplementary Information

**Additional file 1.** Comparison of patient characteristics, diagnostic/examination factors, and pathology factors between the two patient groups delineated based on mammography (MG)-guided procedures for diagnosing DCIS.

**Additional file 2.** Recently reported multiple logistic regression models for ductal carcinoma in situ (DCIS) upstaging.

## Data Availability

The datasets used and/or analyzed during the current study are available from the corresponding author on reasonable request.

## Abbreviations

MG: Mammography
CNB: Core needle biopsy
VAB: Vacuum-assisted biopsy
US: Ultrasonography
US-CNB: Ultrasound-guided core needle biopsy
DCIS: Ductal carcinoma in situ
SLNB: Sentinel lymph node biopsy
NHI: National Health Insurance
BI-RADS: Breast Imaging Reporting and Data System
MG-VAB: MG-guided stereotactic VAB
WLSB: Wire-localized surgical biopsy
ROC: Receiver operating characteristic
BMI: Body mass index
ER: Estrogen receptor
OR: Odds ratio
AUC: Area under the curve

## References

[1] Allred DC (2010). Ductal carcinoma in situ: terminology, classification, and natural history. J Natl Cancer Inst Monogr.

[2] Gradishar WJ, Anderson BO, Abraham J, Aft R, Agnese D, Allison KH (2020). Breast cancer, version 3.2020, NCCN clinical practice guidelines in oncology. J Natl Compr Cancer Netw.

[3] Estourgie SH, Valdés Olmos RA, Nieweg OE, Hoefnagel CA, Rutgers EJT, Kroon BBR (2007). Excision biopsy of breast lesions changes the pattern of lymphatic drainage. Br J Surg.

[4] Coskun G, Dogan L, Karaman N, Ozaslan C, Atalay C (2012). Value of sentinel lymph node biopsy in breast cancer patients with previous excisional biopsy. J Breast Cancer.

[5] Yuan Q, Hou J, Zhou R, Zheng L, Lu F, Deng T (2022). Stepwise limited axillary lymph node dissection based on lymphatic drainage from the breast to decrease breast cancer-related lymphedema: A randomized controlled trial. Ann Surg Oncol.

[6] Brennan ME, Turner RM, Ciatto S, Marinovich ML, French JR, Macaskill P, et al. Ductal carcinoma in situ at core-needle biopsy: Meta-analysis of underestimation and predictors of invasive breast cancer. Radiology. 2011;260:119–28. 10.1148/radiol.11102368 21493791

[7] Park HS, Kim HY, Park S, Kim EK, Kim SL, Park BW (2013). A nomogram for predicting underestimation of invasiveness in ductal carcinoma in situ diagnosed by preoperative needle biopsy. Breast..

[8] Diepstraten SCE, van de Ven SMWY, Pijnappel RM, Peeters PHM, van den Bosch MAAJ, Verkooijen HM, et al. Development and evaluation of a prediction model for underestimated invasive breast cancer in women with ductal carcinoma in situ at stereotactic large core needle biopsy. PLoS One. 2013;8:e77826. 10.1371/journal.pone.0077826 PMC3795649 24147085

[9] Kondo T, Hayashi N, Ohde S, Suzuki K, Yoshida A, Yagata H (2015). A model to predict upstaging to invasive carcinoma in patients preoperatively diagnosed with ductal carcinoma in situ of the breast. J Surg Oncol.

[10] Jakub JW, Murphy BL, Gonzalez AB, Conners AL, Henrichsen TL, Maimone S (2017). A validated nomogram to predict upstaging of ductal carcinoma in situ to invasive disease. Ann Surg Oncol.

[11] Meurs CJC, van Rosmalen J, Menke-Pluijmers MBE, terBraak BPM, de Munck L, Siesling S (2018). A prediction model for underestimation of invasive breast cancer after a biopsy diagnosis of ductal carcinoma in situ: based on 2892 biopsies and 589 invasive cancers. Br J Cancer.

[12] Kim S, Kim J, Park HS, Kim HY, Lee K, Lee J (2019). An updated nomogram for predicting invasiveness in preoperative ductal carcinoma in situ of the breast. Yonsei Med J.

[13] Yen AM, Tsau HS, Fann JC, Chen SL, Chiu SS, Lee YC (2016). Population-based breast cancer screening with risk-based and universal mammography screening compared with clinical breast examination a propensity score analysis of 1 429 890 Taiwanese women. JAMA Oncol.

[14] Silverstein MJ (2003). The University of Southern California/Van Nuys prognostic index for ductal carcinoma in situ of the breast. Am J Surg.

[15] Edge SB, Compton CC. The American Joint Committee on Cancer: The 7th edition of the AJCC cancer staging manual and the future of TNM. Ann Surg Oncol. 2010;17(6):1471–14. 10.1245/s10434-010-0985-4 20180029

[16] Park HS, Park S, Cho J, Park JM, Kim SL, Park BW (2013). Risk predictors of underestimation and the need for sentinel node biopsy in patients diagnosed with ductal carcinoma in situ by preoperative needle biopsy. J Surg Oncol.

[17] Kim J, Han W, Lee JW, You JM, Shin HC, Ahn SK (2012). Factors associated with upstaging from ductal carcinoma in situ following core needle biopsy to invasive cancer in subsequent surgical excision. Breast..

[18] Yuan WH, Hsu HC, Chen YY, Wu CH (2020). Supplemental breast cancer-screening ultrasonography in women with dense breasts: a systematic review and meta-analysis. Br J Cancer.

[19] Suh YJ, Kim MJ, Kim EK, Moon HJ, Kwak JY, Koo HR, et al. Comparison of the underestimation rate in cases with ductal carcinoma in situ at ultrasound-guided core biopsy: 14-Gauge automated core-needle biopsy vs 8- or 11-gauge vacuum-assisted biopsy. Br J Radiol. 2012;85:e349–56. 10.1259/bjr/30974918 PMC3587071 22422382

[20] Philpotts LE, Hooley RJ, Lee CH (2003). Comparison of automated versus vacuum-assisted biopsy methods for sonographically guided core biopsy of the breast. Am J Roentgenol.

[21] Park H, Hong J, Chang SY, Huh JY, Shin JE, Kim J (2015). Differences between the clinical and histopathological tumor stages in breast cancer diagnosed using vacuum-assisted breast biopsy. Oncol Lett.

[22] Tsai HY, Huang ST, Chao MF, Kan JY, Hsu JS, Hou MF, et al. Cost-effectiveness of stereotactic vacuum-assisted biopsy for nonpalpable breast lesions. Eur J Radiol. 2020;127:108982. 10.1016/j.ejrad.2020.108982 32334370

[23] Sung WY, Yang HC, Liao IC, Su YT, Chen FH, Chen SL. Experiences of women who refuse recall for further investigation of abnormal screening mammography: A qualitative study. Int J Environ Res Public Health. 2022;19:1041. 10.3390/ijerph19031041 PMC8834256 35162064

[24] Esen G, Tutar B, Uras C, Calay Z, İnce Ü, Tutar O (2016). Vacuum-assisted stereotactic breast biopsy in the diagnosis and management of suspicious microcalcifications. Diagn Interv Radiol.

[25] Huang CS, Wu CY, Chu JS, Lin JH, Hsu SM, Chang KJ (1999). Microcalcifications of non-palpable breast lesions detected by ultrasonography: Correlation with mammography and histopathology. Ultrasound Obstet Gynecol.

