# Supplementary material for: High rate of postoperative upstaging of ductal carcinoma in situ when prioritizing ultrasound evaluation of mammography-detected lesions: a single-center retrospective cohort study
Source: World J Surg Oncol. 2023 Feb 17;21:48. doi: 10.1186/s12957-023-02900-6 (PMC9936646; doi:10.1186/s12957-023-02900-6)
Supplement: Supplementary file 1 — Additional file 1. Comparison of patient characteristics, diagnostic/examination factors, and pathology factors between the two patient groups delineated based on mammography (MG)-guided procedures for diagnosing DCIS. [file 12957_2023_2900_MOESM1_ESM.docx]

**Additional file 1. Comparison of patient characteristics, diagnostic/examination factors, and pathology factors between the two patient groups delineated based on mammography (MG)-guided procedures for diagnosing DCIS**

| **Variables** | ***n*** | **ST-VAB (*n* = 31)** | **WLSB (*n* = 41)** | ***P^a^*** |
| --- | --- | --- | --- | --- |
| **Patient characteristics** |  |  |  |  |
| Age (years) | 72 |  |  | 0.14 |
| <50 |  | 7 | 16 |  |
| ≥50 |  | 24 | 25 |  |
| BMI (kg/m^2^) | 72 |  |  | 0.39 |
| <23.5 |  | 15 | 24 |  |
| ≥23.5 |  | 16 | 17 |  |
| Previous history of breast cancer | 72 |  |  | >0.99 |
| No |  | 28 | 37 |  |
| Yes |  | 3 | 4 |  |
| Family history of breast cancer | 64 |  |  | 0.20 |
| No |  | 19 | 31 |  |
| Yes |  | 8 | 6 |  |
| Lesion side | 72 |  |  | 0.56 |
| Left |  | 18 | 21 |  |
| Right |  | 13 | 20 |  |
| **Diagnostic/Examination factors** |  |  |  |  |
| Initial detection method | 72 |  |  | 0.43 |
| Screening imaging |  | 30 | 41 |  |
| Palpable lesion |  | 0 | 0 |  |
| Nipple discharge |  | 1 | 0 |  |
| Lesion under US | 61 |  |  | >0.99 |
| Not detectable |  | 26 | 32 |  |
| Detectable |  | 1 | 2 |  |
| MG BI-RADS category | 71 |  |  | 0.32 |
| 3 |  | 1 | 0 |  |
| 4 |  | 30 | 39 |  |
| 5 |  | 0 | 2 |  |
| MG mass-associated findings | 67 |  |  | 0.004 |
| No |  | 29 | 29 |  |
| Yes |  | 0 | 9 |  |
| **DCIS-associated factors** | 61 |  |  |  |
| DCIS grade |  |  |  | 0.69 |
| Low |  | 8 | 6 |  |
| Intermediate |  | 13 | 15 |  |
| High |  | 8 | 11 |  |
| Suspicious of microinvasion | 71 |  |  | (NA) |
| No |  | 31 | 40 |  |
| Yes |  | 0 | 0 |  |
| ER | 57 |  |  | 0.68 |
| Negative |  | 3 | 3 |  |
| Positive |  | 20 | 31 |  |
| **Final staging results** | 72 |  |  | 0.65 |
| DCIS |  | 28 | 39 |  |
| Invasive cancer (upstage %) |  | 3 (9.7) | 2 (4.9) |  |
| *^a^*Fisher’s exact test  Abbreviations: US-CNB, ultrasound-guided core needle biopsy; ST-VAB, stereotactic vacuum-assisted breast biopsy; WLSB, wire-localized surgical biopsy; US, ultrasound; MMG, mammogram; BI-RADS, The Breast Imaging and Data System. | | | | |
