# Supplementary material for: High rate of postoperative upstaging of ductal carcinoma in situ when prioritizing ultrasound evaluation of mammography-detected lesions: a single-center retrospective cohort study
Source: World J Surg Oncol. 2023 Feb 17;21:48. doi: 10.1186/s12957-023-02900-6 (PMC9936646; doi:10.1186/s12957-023-02900-6)
Supplement: Supplementary file 2 — Additional file 2. Recently reported multiple logistic regression models for ductal carcinoma in situ (DCIS) upstaging. [file 12957_2023_2900_MOESM2_ESM.zip › 1-Additional_file_2.docx]

**Additional file 2. Recently reported multiple logistic regression models for ductal carcinoma in situ (DCIS) upstaging**

| **Author (Year)** | **Biopsy method** | ***N*** | **Predictive factors** | **AUC** |
| --- | --- | --- | --- | --- |
| Park, H. S.  (2013) [7] | US-CNB  US-VAB  ST-VAB | 330 | Suspicious microinvasion, biopsy method (CNB), US calcification, palpability, US mass | 0.75 |
| Diepstraten, S. C. E.  (2013) [8] | ST-CNB | 348 | Lobular cancerization, microinvasion, core numbers, lesion size | 0.66 |
| Kondo, T.  (2015) [9] | Unknown | 1187 | Pleomorphic calcifications, suspicious on US or MRI, size ≥ 2 cm (MRI), Her-2 positive, comedo necrosis, no sclerosing adenosis | 0.69 |
| Jakub, J. W.  (2017) [10] | CNB/VAB  (pooled US, ST, MRI) | 834 | High grade, mass lesion on imaging, large linear dimension | 0.71 |
| Meurs, C. J. C.  (2018) [11] | Unknown | 3281 | Age, detection mode, palpability, BI-RADS score, DCIS grade, suspected microinvasion | 0.69 |
| Kim, S.  (2019) [12] | US-CNB,  ST-VAB | 444 | BCT, Her-2(+), comedo necrosis, US mass, MG mass, suspicious microinvasion | 0.62 |
| Abbreviations: AUC, area under curve; ST, stereotaxis; US, ultrasound; MRI, magnetic resonance imaging; CNB, core needle biopsy; VAB, vacuum-assisted breast biopsy; DCIS, ductal carcinoma in situ; Her-2, human epidermal growth factor receptor 2; BCT, breast-conserving therapy | | | | |
